# Incidental Follow-up Imaging of Previous Ventricular Tuberculosis and Pneumoencephalography in a 57-year-old man

**DOI:** 10.7759/cureus.6340

**Published:** 2019-12-10

**Authors:** Sandra Neumann, Matthew D Kobetić

**Affiliations:** 1 Neurology, Bristol Medical School, University of Bristol, Bristol, GBR; 2 Surgery, Bristol Medical School, University of Bristol, Bristol, GBR

**Keywords:** ventricular tuberculosis, tuberculosis, mri, central tuberculosis, pneumoencephalography, magnetic resonance imaging

## Abstract

We present a rare case of follow-up by neuroimaging in a 57-year-old man with a previous pneumoencephalography to evaluate ventricular tuberculosis (TB). Magnetic resonance imaging (MRI) of the whole head was performed at 3T using T1‐weighted magnetization‐prepared rapid gradient echo (T1‐MPRAGE). A full quantitative sensory testing battery on the forearm was also performed, alongside a brief clinical examination. All test results were normal with the exception of the T1-MPRAGE which showed enlarged ventricles and a cyst-like focal changes, mistaken for a sign of old ischaemic infarct. The change, however, is consistent with the insertion of a cannula for the pneumoencephalogram. This is the first follow-up report with neuroimaging presented nearly 40 years after the diagnosis of ventricular TB.

## Introduction

Tuberculosis (TB) is an infectious disease usually caused by Mycobacterium tuberculosis. In rare cases, TB may migrate into the central nervous system, which presents the most severe form of TB. One complication of TB in the central nervous system is tubercular ventriculitis, which remains an under-recognised complication [1]. Thus, the literature on the occurrence, pathophysiology, treatment, and prognosis is limited.

The diagnosis of tubercular ventriculitis is typically done through magnetic resonance imaging (MRI) [2], with the most common presenting features of hydrocephalus, ependymal enhancement of the lateral and/or fourth ventricle, occasionally with restricted diffusion, and meningeal enhancement [1-2]. Despite the use of MRI, there is no agreed-upon protocol for best practice.

Typically, tubercular ventriculitis is treated by anti-TB treatments, cerebrospinal fluid (CSF) diversion, for example, ventriculoperitoneal shunt, and occasionally with the addition of other surgical interventions such as temporal cranioectomy or temporal lobectomy, although operative interventions as such are often restricted to cases also presenting with seizures [2-3]. Patients may be followed up in clinics, but are rarely followed up with MRI or other types of imaging; therefore, the long-term neuropathological impact of ventriculitis remains unknown.

We report the first follow-up of a 57-year-old man who suffered tubercular ventriculitis aged 16.

## Case presentation

A 57-year-old man took part in a research trial at the Clinical Research and Imaging Centre, Bristol, United Kingdom. The man was screened for pre-existing conditions including musculoskeletal pain, cardiovascular, respiratory, psychiatric, immunological, oncological, urogenital, gastrointestinal, endocrine, neurological, rheumatologic and ophthalmic disorders. Importantly, the medical history was only taken with respect to on-going diagnoses with the exception of oncological disorders. In addition, a 12-lead electrocardiogram (ECG), clinic and ambulatory blood pressure and full-screen sensory testing on the forearm was performed for research purposes, which were all confirmed to be within the normal range.

**Figure 1:**
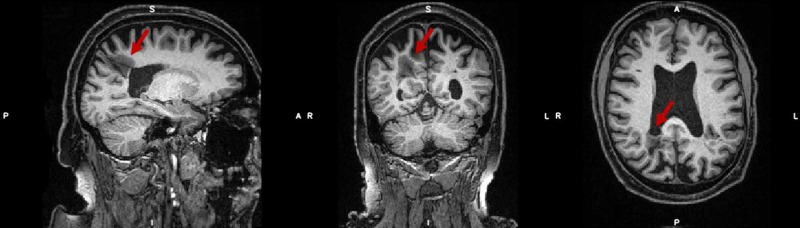
MRI scan (T1-MPRAGE) performed at 3T during research visit The hypo-intense area in the right hemisphere (indicated by the red arrows) measures approximately 13 x 20 x 26 mm (x, y, z). MRI: magnetic resonance imaging; T1‐MPRAGE: T1‐weighted magnetization‐prepared rapid gradient echo.

During the research MRI scan, a T1‐weighted magnetization‐prepared rapid gradient echo (T1‐MPRAGE) of the whole head was obtained (Figure 1). This showed confluent low intensity in the right parietal white matter containing a focal cystic change. The preliminary neuroradiology report noted generally reduced volume compared to that expected for the patient’s age.

**Figure 2:**
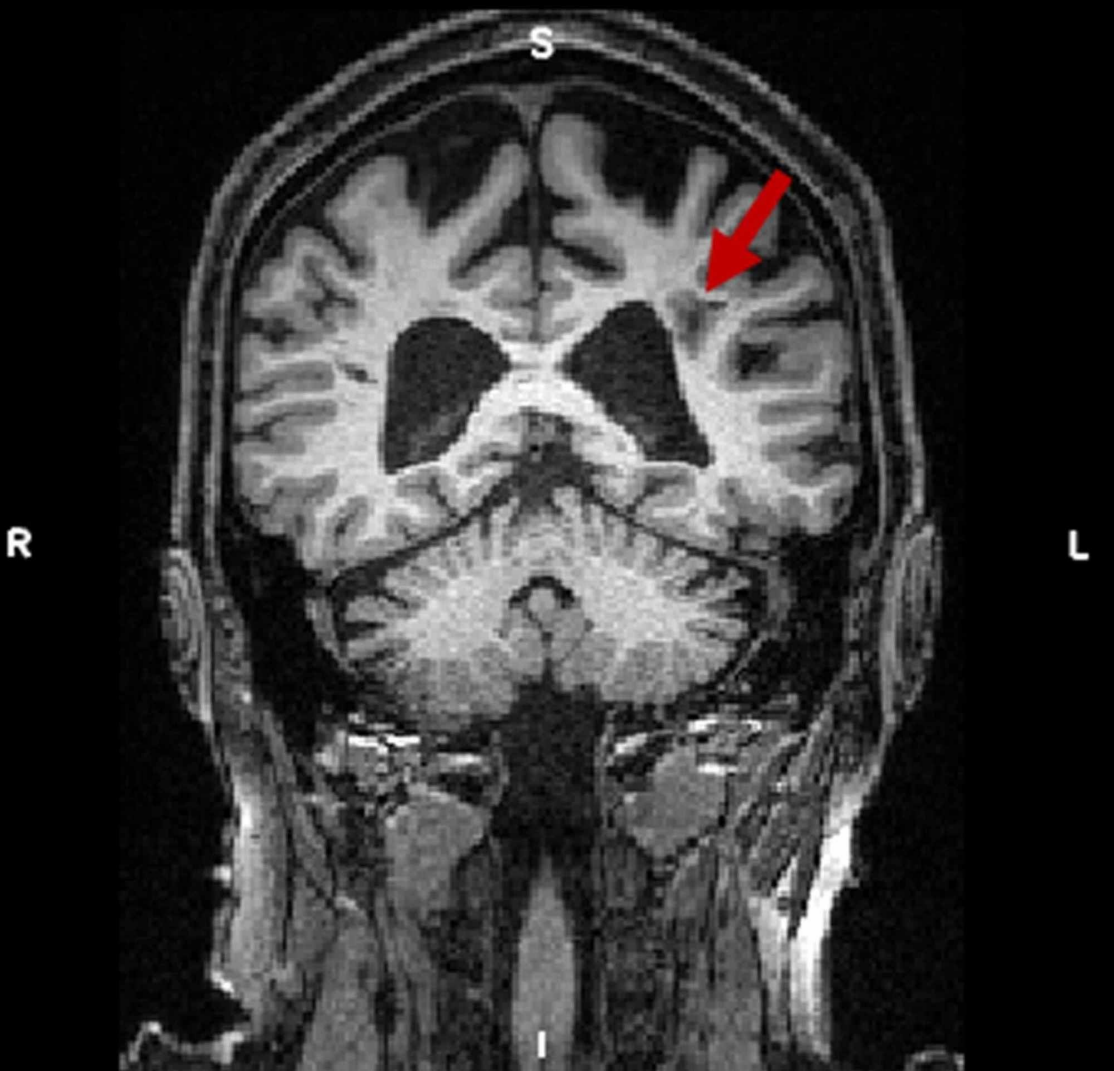
Hypointensity observed in the left hemisphere The hypointensity is indicated by the left arrow. Note also the relative enlargement of the ventricles.

A less prominent hypointensity was observed in the left hemisphere (Figure 2).

**Figure 3:**
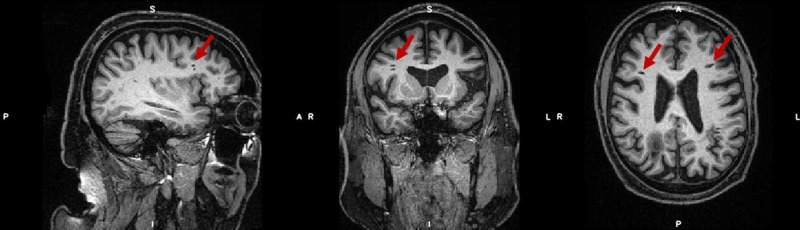
MRI scan (T1-MPRAGE) performed at 3T during research visit Position shows the smaller focal lesions in the frontal lobe as indicated by the red arrows. MRI: magnetic resonance imaging; T1‐MPRAGE: T1‐weighted magnetization‐prepared rapid gradient echo.

In addition to the main focal lesion in the parietal white matter, at least three smaller focal lesions were seen in the frontal lobe white matter, two in the right hemisphere and one at a similar lateral and frontal position in the left hemisphere (Figure 3).

Based on a preliminary report of the T1-MPRAGE, it was recommended that the patient be followed up in the Stroke Clinic as the suspected lesion was thought to represent small vessel ischaemia possibly with lacunar infarct.

The patient was invited for a clinical MR scan through a stroke clinic in the South of England. This scan showed bilateral susceptibility artefact lateral to the third ventricle, bilateral signal change adjacent the posterior horn of the lateral ventricles, prominent lateral ventricles, and linear signal voids extending to the cortical surface, with overlying cortical defects. The clinician noted that this was likely the site of previous craniotomy or intervention.

The patient was followed up by consultation, which revealed that he had suffered tubercular ventriculitis at age 16 and had been admitted to the Midland Centre for Neurosurgery and Neurology. At the time, pneumoencephalography was performed to assess the patient, which resulted in a coma lasting approximately one week, after which the patient recovered with anti-TB treatment.

## Discussion

No previous reports have shown follow-up MR of patients with tubercular ventriculitis; nor have we found reports of cases followed up 40 years after pneumoencephalography, a procedure known to carry a high risk of organic cause sequalae [4]. In the present case report, the sequelae of the procedure (i.e. the coma immediately following the procedure) resolved within one week, and the patient made a full recovery.

One report on the use of pneumoencephalography in children with tuberculous meningitis showed that of 26 children with abnormal anatomy only eight were alive at the 11-month follow up, and of these, all patients showed neurological or mental residual sequelae [5]. Unfortunately, we were unable to source the original pneumoencephalogram due to the closure of the Midland Centre for Neurosurgery and Neurology. For this reason, it cannot be definitively ascertained that the lesions are consistent with those of the pneuomencephalographic procedure. On balance, however, the large lesions in the parietal cortex are most likely explained by this procedure and are consistent with the placement of a shunt-like object into the ventricular space.

In the present case report, the patient showed several large lesions in the parietal cortex, as well as some lesions in the frontal lobe and enlarged ventricles. Interestingly, there were no detectable sequelae; the patient presented with no signs or symptoms suggestive of the presence of such anatomical lesions. Whilst the parietal lesions are consistent with the placement of a shunt for the pneumoencephalography, it is interesting that several smaller lesions are present which could indicate organic injury as a consequence of central nervous system TB. However, as a differential diagnosis, it cannot be ruled out that the smaller lesions (as seen in Figure 3) are due to cerebral small vessel disease. Here, it is worth noting that the patient was non-diabetic, and had a normal resting 24-hour ambulatory blood pressure. 

The present incident also demonstrates the case for comprehensive history taking in otherwise healthy patients prior to imaging for research purposes. This is emphasised by the initial report mistakenly suggestive of old ischaemic infarct, which could have been ruled out had the history of the pneumoencephalographic procedure been known.

## Conclusions

The present case is a unique incidental follow-up of ventricular TB in a patient who previously had invasive pneumoencephalography at the age of 16. We present a curious case of a relatively large lesion to the parietal cortex with no documented functional impairment. The patient in question has achieved high academic honours, and present with no discernible neurological deficits.
